# *GmNMHC5* Modulates Gibberellin Homeostasis to Balance Carbon–Nitrogen Metabolism and Enhance Protein Yield in Soybean

**DOI:** 10.1002/fsn3.70659

**Published:** 2025-08-07

**Authors:** Xinlei Chen, Wenwen Song, Zhongfa Zhang, Chenchen Zhou, Peihang Wu, Shujun Wang, Shi Sun, Yupeng Zhu, Cailong Xu, Cunxiang Wu

**Affiliations:** ^1^ Key Laboratory of Soybean Biology of Ministry of Education China Northeast Agricultural University Harbin China; ^2^ Key Laboratory of Soybean Biology (Beijing), Ministry of Agriculture and Rural Affairs Institute of Crop Sciences, Chinese Academy of Agricultural Sciences Beijing China

**Keywords:** gibberellin, *GmNMHC5*, quality, soybean, yield

## Abstract

Soybean, a vital source of high‐quality plant‐based protein and oil, continues to be a focal crop for improving yield and quality. Gibberellins (GAs), as key regulators of plant growth and development, hold significant potential for enhancing these traits in soybean. In this study, previously developed *GmNMHC5* mutant lines were utilized to assess GA levels, photosynthetic capacity, carbohydrate and nitrogen accumulation, as well as yield and quality characteristics during the seed‐filling stage. Knockout of *GmNMHC5* was found to enhance endogenous GA biosynthesis, resulting in increased plant height and improved carbon and nitrogen metabolism. These physiological changes contributed to significant increases in both seed weight and size, leading to higher accumulation of carbohydrate and protein reserves in mature seeds. Although total seed yield remained unchanged, protein yield per plant was significantly elevated in the *GmNMHC5* knockout line. In contrast, overexpression of *GmNMHC5* led to reduced GA levels, decreased plant height, and diminished aboveground dry matter accumulation, ultimately lowering protein yield. These findings indicate that targeted knockout of *GmNMHC5* can enhance protein yield in soybean by modulating GA levels and optimizing carbon–nitrogen allocation. This study provides a theoretical basis and valuable genetic resources for the development of high‐protein soybean cultivars.

## Graphical abstract

The *GmNMHC5* knockout elevated gibberellin (GA) biosynthesis, resulting in increased plant height and greater accumulation of carbohydrates and proteins in mature seeds, thereby enhancing protein yield per plant without altering total yield. Conversely, *GmNMHC5* overexpression lines exhibited reduced GA levels, suppressed plant growth, and decreased aboveground dry matter accumulation, ultimately resulting in lower protein yield. These results suggest that *GmNMHC5* knockout enhances soybean protein yield by fine‐tuning GA homeostasis and optimizing carbon–nitrogen allocation.

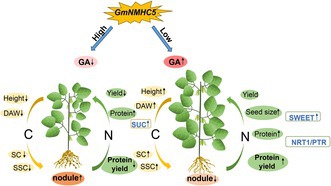

## 1 Introduction

Soybean is a critical source of high‐quality edible plant oil and protein for both human and animal consumption (Li, Sun, et al. 2024). Soybean cultivation exhibits a well‐documented negative correlation between seed protein concentration and yield output, primarily due to resource allocation constraints during seed development. This physiological trade‐off creates a persistent breeding challenge in simultaneously improving nutritional quality and agricultural productivity (Cui et al. 2025). The seed‐filling stage plays an important role in governing soybean yield–quality determination. During this period, photoassimilates are transported from source leaves to developing seeds, establishing the biochemical basis for nitrogen assimilation and storage compound deposition (Saldivar et al. 2011). The metabolic balance between carbon and nitrogen (C–N) directly modulates the allocation of resources toward protein versus oil biosynthesis (Kambhampati et al. 2021). Increasing evidence reveals that phytohormones, as central regulators of C–N metabolic coordination, determine source–sink relationships by synchronizing photosynthetic activity with nitrogen transporter dynamics (Nguyen et al. 2016; Zhang et al. 2021). This metabolic–hormonal interaction provides a systemic framework for manipulating C–N resource trade‐offs to mitigate the yield–quality antagonism. Gibberellins (GAs), as the most structurally diverse class of phytohormones, comprise 136 identified variants across plants, fungi, and bacteria. Notably, only four bioactive forms (GA_1_, GA_3_, GA_4_, GA_7_) directly regulate physiological processes in plants, with the majority functioning as biosynthetic intermediates or inactive catabolites (Barnes 2014). These bioactive GAs orchestrate pivotal developmental transitions through dose‐dependent modulation of seed germination, hypocotyl elongation, leaf expansion, and seed development (Jing et al. 2024; Li, Zhou, et al. 2024; Taniguchi et al. 2018). In major crops, GA‐mediated signaling pathways demonstrate spatiotemporal specificity in coordinating C–N partitioning during flowering and seed maturation, thereby influencing final grain yield and quality (Hu et al. 2022). Within carbon metabolism, GAs modulate the distribution of photoassimilates through architectural remodeling; their signaling cascades precisely govern internode cell elongation, thereby determining crop height phenotypes (Zhou et al. 2023). Overexpression of the GA‐responsive gene *OsGASR9* in rice enhances lodging resistance and increases grain yield (Li et al. 2019), whereas CRISPR‐mediated knockout of soybean *GmGA3ox1* elevates photosynthetic carbon assimilation efficiency and boosts biomass accumulation (Chu et al. 2022). Concurrently, GA‐activated sucrose transporters (SWEETs) optimize carbon flux partitioning by directing photoassimilate translocation toward developing seeds (Wang, Xue, et al. 2022). Regarding nitrogen metabolism, GAs exhibit dual regulatory capacities. First, they dynamically regulate nodulation gene expression to modulate soybean root nodule density and nitrogen fixation efficiency (Sun et al. 2021). Second, GA signaling pathways coordinate with nitrogen transporters such as ammonium transporter *AMT1* and nitrate transporter *NRT2.1* to reprogram nitrogen remobilization toward reproductive organs, thus improving nitrogen assimilation efficiency (Sun et al. 2021; Wang, Yao, et al. 2020).

Our previous work identified GmNMHC5, a MADS‐box transcriptional regulator that interacts with the GA signaling repressor GmGAI to coordinate flowering initiation and nodulation in soybean (Liu et al. 2015; Wang, Wang, et al. 2020, 2022). However, its potential role in both mediating GA‐regulated C–N metabolic networks during seed filling and consequently influencing the critical yield–quality trade‐off remains unexplored. This knowledge gap motivated our current investigation into how *GmNMHC5* synchronizes sucrose allocation and nitrogen remobilization during reproductive development to ultimately determine seed composition and yield potential.

## 2 Materials and Methods

### 2.1 Plant Materials and Growth Conditions

The soybean materials utilized in this study comprised the variety of Jack as wild‐type (WT), a *CRISPR‐Cas9*‐mediated *GmNMHC5* knockout line (*Gmnmhc5*), and two *GmNMHC5* overexpression lines (OE*GmNMHC5*‐#25 and OE*GmNMHC5*‐#32). These materials were developed and preserved by our group at the Institute of Crop Sciences, Chinese Academy of Agricultural Sciences (Wang, Wang, et al. 2022). The relative expression of *GmNMHC5* in WT and overexpression lines was determined using a reverse transcription quantitative polymerase chain reaction (RT‐qPCR) method and shown as Figure S1. The potted experiments were conducted in 2022 and 2023 using pots measuring 24 cm in height and 21 cm in diameter, filled with soil to a depth of 20 cm. The soil, collected locally from the 0–20 cm plow layer, served as the growth medium. In each pot, 10 uniformly sized soybean seeds were sown and covered with approximately 2 cm of soil during the summer. Once the trifoliate leaves had fully expanded, 5 uniform and well‐grown plants per pot were retained. The physiological traits were evaluated in 2024 (Figure S2); field experiments were carried out with each plot containing three rows of each soybean line (WT, *Gmnmhc5*, OE*GmNMHC5*‐#25, and OE*GmNMHC5*‐#32), with each row measuring 6 m in length. Direct seeding was employed at a uniform density, and a protective row was planted between experimental rows. All plants were grown under natural light and temperature conditions until maturity. Physiological traits were evaluated during the 2022–2023 growing seasons, while yield and quality parameters were analyzed across three consecutive years (2022–2024) to ensure data robustness and reproducibility.

### 2.2 Measurement of GAs

Leaves, pod walls, and seeds from three individual plants of WT, *Gmnmhc5*, OE*GmNMHC5*‐#25, and OE*GmNMHC5*‐#32 were sampled at the seed‐filling stage (R5.5). The samples were frozen in liquid nitrogen and stored at −80°C in an ultra‐low temperature freezer. GA contents were measured by Metware Biotechnology Co. Ltd. (Wuhan, China) using a UPLC‐ESI‐MS/MS system. Three independent experiments were performed.

### 2.3 Measurement of Leaf Photosynthesis and SPAD

At the R5.5 stage, between 9:00 and 11:00 AM on sunny days, photosynthetic performance was measured using a LI‐6400 portable photosynthesis system (LI‐COR, USA) with the following parameters set: light quantum flux at 1200 μmol/(m^2^ s), CO_2_ concentration at 450 μmol/m^2^, flow rate at 500 μmol/s, and temperature at 25°C. The assessed parameters included net photosynthetic rate (Pn), transpiration rate (Tr), stomatal conductance (Gs), and intercellular CO_2_ concentration (Ci). Leaves with uniform growth, selected from the same position on the plant, were used for measurement. The SPAD value of functional leaves (the third leaf from the top) was measured using a SPAD‐502PLUS chlorophyll meter (Konica Minolta, Japan).

### 2.4 Measurement of Leaf Area, Plant Height, and Dry Matter

Three individual plants of WT, *Gmnmhc5*, OE*GmNMHC5*‐#25, and OE*GmNMHC5*‐#32 were selected randomly at the R5.5 stage for the measurement of leaf area, plant height, and dry matter. Leaf area was quantified by a portable leaf area meter (Hangmei, China). Plant height was determined by measuring the distance from the cotyledonary node to the terminal growth point with a soft ruler. After separation into leaves, petioles, stem, pod walls, and seeds, all samples placed in kraft paper bags were subjected to 105°C oven‐drying for 30 min and then maintained at 70°C until reaching constant mass before final weighing. Three independent experiments were performed.

### 2.5 Measurement of Soluble Sugar and Starch Content

Dried samples were ground and passed through a 100‐mesh sieve. A 0.01 g subsample was then transferred to a 2 mL centrifuge tube, and the soluble sugar and starch contents were quantified using an assay kit (Solarbio, China) following the manufacturer's protocol.

### 2.6 Measurement of Soybean Seed Morphology

Seed length, width, and thickness were quantified using a digital vernier caliper (Mitutoyo, Kawasaki, Japan; precision ±0.01 mm) following a randomized sampling design. For each genotype, three biological replicates consisting of 30 seeds per replicate were subjected to morphometric analysis. Seed morphological characteristics were documented using a standardized imaging system (Canon EOS 90D with 100 mm macro lens) under controlled lighting conditions (5000 K).

### 2.7 Measurement of Protein and Oil Content in Seeds

Mature seeds were harvested and subjected to protein and oil composition analysis using a Near‐Infrared Spectrophotometer (Infratec 1255 Grain Analyzer, FOSS, Denmark). Three technical replicates were performed for each biological sample to ensure measurement reproducibility.

### 2.8 Yield and Yield Components

For yield assessment, 15 plants from each line were selected. Measurements included 100‐seed weight, number of pods and seeds per plant, plant height, stem diameter, number of nodes on the main stem, first pod height, and internode length.

### 2.9 RNA‐Seq and Data Analysis

Seeds from three individual plants of WT and *Gmnmhc5* were sampled at R5.5 stage. The collected seeds were flash‐frozen in liquid nitrogen, and stored at −80°C until further processing. Subsequently, the samples were entrusted to Metware Biotechnology Co. Ltd. (Wuhan, China) for RNA extraction, purification, and library construction. Next Generation Sequencing (NGS) technology was employed, and sequencing was performed on the Illumina platform using paired‐end (PE) sequencing. Three independent experiments were performed. The raw sequencing data were filtered, and the resulting high‐quality sequences were aligned to the reference genome of the species. Differentially expressed genes (DEGs) between WT and *Gmnmhc5* were identified using DESeq2, with a significance threshold of |log_2_FC| > 1 and adjusted *p* < 0.05. DEGs were annotated and functionally enriched using Gene Ontology (GO) and Kyoto Encyclopedia of Genes and Genomes (KEGG) databases to identify key pathways and biological processes.

### 2.10 RT‐qPCR Analysis

RNA was isolated as previous study (Wang, Wang, et al. 2022; Wang, Xue, et al. 2022), and total RNA was reverse‐transcribed into first‐strand cDNA using the Hifair AdvanceFast 1st Strand cDNA Synthesis Kit (Yeasen Biotechnology, Shanghai, China). RT‐qPCR amplification was performed using Tag Pro Universal SYBRgPCR Master Mix (TIANGEN, Beijing, China) on an Applied Biosystems TM 7500 Fast Dx Real‐Time PCR Instrument (ABI, Foster City, CA, USA). The PCR cycling conditions were as follows: 95°C for 10 min, then 40 cycles of 95°C for 10 s, 60°C for 20 s, and 72°C for 30 s. *GmActin* was used as an endogenous regulatory gene for soybean. Three biological replicates and three technical replicates were used for each assay. The nucleotide sequences of the primers used in the study were listed in Table S1.

### 2.11 Statistical Analyses

Data entry and calculations were carried out using Microsoft Excel, while statistical analysis was conducted using SPSS software. As the results for physiological traits were consistent between the years of 2022 and 2023, the two‐year average was used for analysis. Additionally, the quality and yield trait data from each year were analyzed separately. Graphs were generated using Origin 2021 software. Statistical significance was defined as *p < 0.05, **p < 0.01, ***p < 0.001.

## 3 Results

### 3.1 *GmNMHC5* Negatively Regulates the Accumulation of GA in Leaves, Pod Walls, and Seeds

#### 3.1.1 The Content and Composition of GAs in Leaves

**FIGURE 1:**
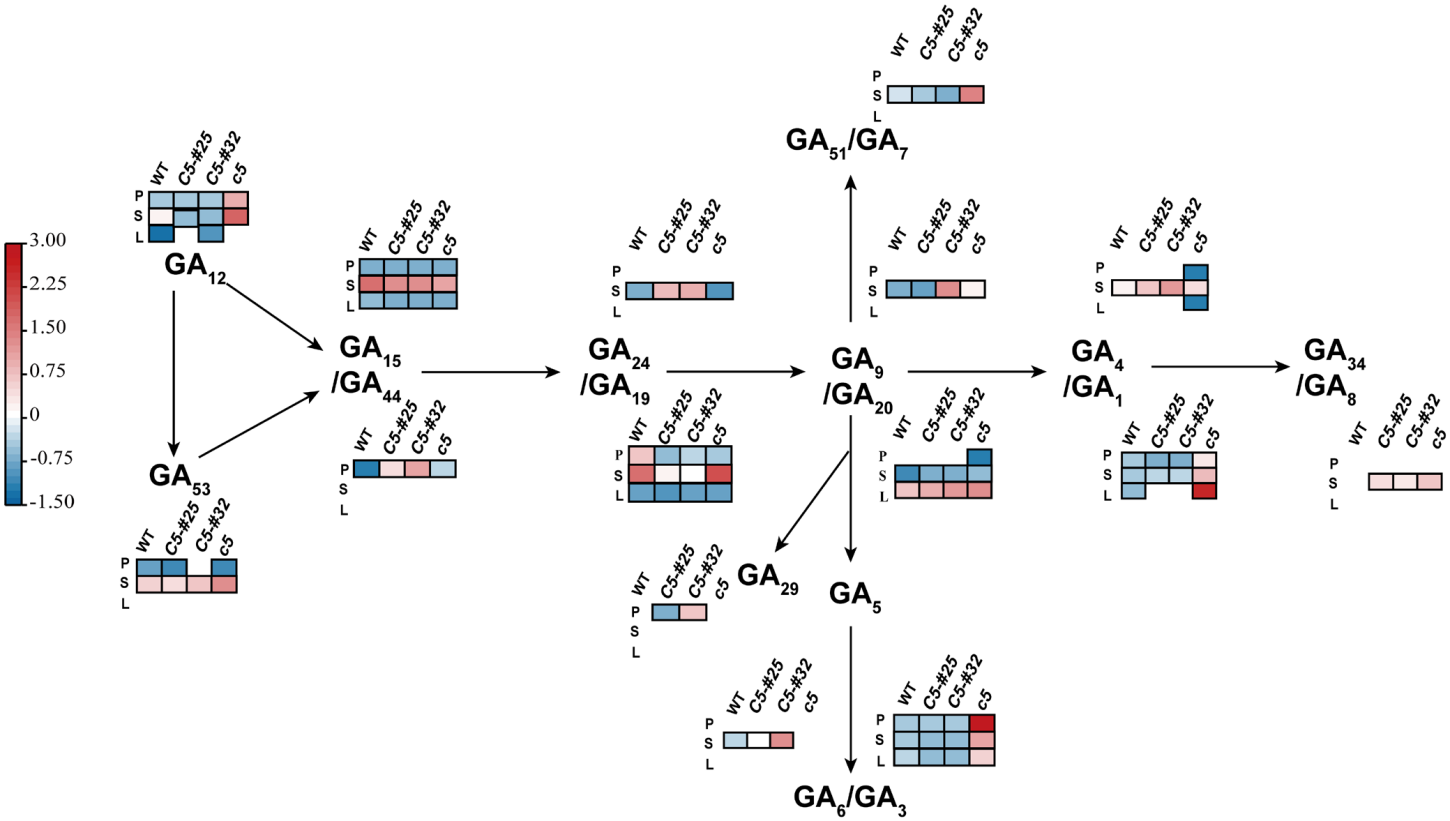
Unified GA biosynthetic pathway with a heatmap showing GA content in leaves (L), pod walls (P), and seeds (S) of WT, *Gmnmhc5* (*c5*), OE*GMNMHC5*‐#25 (*C5‐#25*), and OE*GMNMHC5*‐#32 (*C5‐#32*), where red and blue indicate high and low GA content.

Hormonal profiling revealed distinct GA composition patterns in leaf tissues, with GA_1_ and GA_3_ identified as the predominant bioactive forms, accompanied by GA_15_, GA_19_, and GA_20_ as minor components (Table S2, Figure 1). Quantitative analysis demonstrated significant GA depletion in *GmNMHC5* overexpression lines, showing 83% and 78% reductions in total GA content for *OEGmNMHC5*‐#25 and *OEGmNMHC5*‐#32, respectively, predominantly attributed to diminished GA_1_ (72% decrease) and GA_3_ (88%–90% decrease) levels compared to the WT. Conversely, Gmnmhc5 leaves exhibited a 3.85‐fold upregulation in total GA accumulation, with GA_1_ and GA_3_ showing 2.5‐fold and 4.42‐fold increases, respectively, indicating a negative regulatory role of GmNMHC5 in GA biosynthesis.

#### 3.1.2 The Content and Composition of GAs in Seeds

A total of 12 GA‐related components were detected in soybean seeds, comprising four bioactive forms (GA_1_, GA_3_, GA_4_, GA_7_) and eight biosynthetic precursors/intermediates (GA_9_, GA_12_, GA_15_, GA_19_, GA_20_, GA_24_, GA_44_, GA_53_). *GmNMHC5* overexpression lines exhibited significant GA depletion, with total GA content reduced by 19% (OE*GmNMHC5*‐#25) and 9% (OE*GmNMHC5*‐#32) relative to the WT, predominantly attributed to diminished GA_19_ (59%–64% reduction) and GA_3_ (57%–60% reduction) levels. Conversely, *Gmnmhc5* seeds displayed a 1.71‐fold upregulation in total GA accumulation, driven primarily by elevated GA_1_ (3.7‐fold), GA_3_ (13.44‐fold), and GA_53_ (0.45‐fold) concentrations. These findings demonstrated that *GmNMHC5* functioned as a negative regulator of GA biosynthesis during seed development.

#### 3.1.3 The Content and Composition of GAs in Pod Walls

The composition of GAs in soybean pod walls varied among the different lines. The WT plants predominantly accumulated GA_1_, GA_3_, GA_19_, and GA_53_, whereas *GmNMHC5* overexpression lines showed altered profiles dominated by GA_3_, GA_19_, and GA_29_. *Gmnmhc5* maintained GA_1_, GA_3_, and GA_19_ as major components but with significantly elevated concentrations. Quantitative analysis revealed 57% and 61% reductions in total GA content in OE*GmNMHC5*‐#25 and OE*GmNMHC5*‐#32, respectively, compared to the WT. These decreases were primarily driven by the depletion of GA_3_ (10%–35% reduction) and GA_19_ (66%–78% reduction). Conversely, the *Gmnmhc5* mutant exhibited a 7.16‐fold increase in total GA content, with GA_1_ and GA_3_ showing dramatic upregulation (14.89‐fold and 19.14‐fold, respectively), indicating a pivotal role of *GmNMHC5* in modulating GA homeostasis during pod wall development.

### 3.2 Effects of the *GmNMHC5* on Soybean Yield and Yield Components

Multi‐year yield analysis demonstrated that *GmNMHC5* significantly modulates soybean productivity (Table 1). Overexpression lines *OEGmNMHC5*‐#25 and *OEGmNMHC5*‐#32 exhibited 40% and 16% reductions in average single‐plant yield, respectively, primarily attributed to decreased pod number (40% and 26% reductions) and seed number per plant (38% and 22% reductions) across three growing seasons, while 100‐seed weight remained statistically invariant. In contrast, *Gmnmhc5* plants maintained comparable total yields to the WT despite 14% and 7% reductions in pod and seed number, respectively, due to a compensatory 19% increase in 100‐seed weight.

**TABLE 1:** Effects of the *GmNMHC5* on soybean yield and yield components in three growing seasons (2022–2024).

Year	Cultivar	Pods per plant	Seeds per plant	100‐seed weight (g)	Yield (g plant^−1^)
2022	WT	28.60 ± 1.83 a	52.46 ± 3.03 a	13.55 ± 0.32 c	7.91 ± 0.37 a
	OE*GmNMHC5*‐#25	14.50 ± 1.26 b	27.20 ± 3.43 b	14.40 ± 0.47 bc	4.23 ± 0.44 b
	OE*GmNMHC5*‐#32	18.00 ± 1.94 b	35.82 ± 3.66 b	15.42 ± 0.51 b	5.72 ± 0.61 b
	*Gmnmhc5*	26.10 ± 1.78 a	49.40 ± 2.07 a	16.92 ± 0.38 a	8.46 ± 0.53 a
2023	WT	31.07 ± 1.62 a	61.75 ± 2.25 a	13.41 ± 0.20 c	8.28 ± 0.30 a
	OE*GmNMHC5*‐#25	24.92 ± 1.96 b	53.25 ± 1.65 b	13.90 ± 0.06 bc	7.40 ± 0.23 b
	OE*GmNMHC5*‐#32	27.38 ± 1.74 ab	55.25 ± 1.49 b	14.32 ± 0.06 b	7.91 ± 0.21 ab
	*Gmnmhc5*	24.92 ± 2.07 b	53.00 ± 0.71 b	15.33 ± 0.24 a	8.13 ± 0.11 a
2024	WT	76.30 ± 4.22 a	164.67 ± 8.73 a	14.41 ± 0.11 c	26.40 ± 2.01 ab
	OE*GmNMHC5*‐#25	37.00 ± 2.91 c	80.78 ± 7.65 c	14.51 ± 0.12 c	10.13 ± 1.21 c
	OE*GmNMHC5*‐#32	53.50 ± 2.88 b	126.33 ± 12.74 b	15.29 ± 0.15 b	22.25 ± 2.27 b
	*Gmnmhc5*	65.88 ± 3.47 a	164.00 ± 8.14 a	16.32 ± 0.12 a	29.19 ± 1.32 a

*Note:* Values with the same letter are not different at *p* < 0.05 within the same parameter. Significance analysis of yield components was performed among WT and *GmNMHC*5 mutant lines within the same year.

### 3.3 Effects of *GmNMHC5* on Soybean Plant Height, Internode Length, and Lower First Pod Height

**FIGURE 2:**
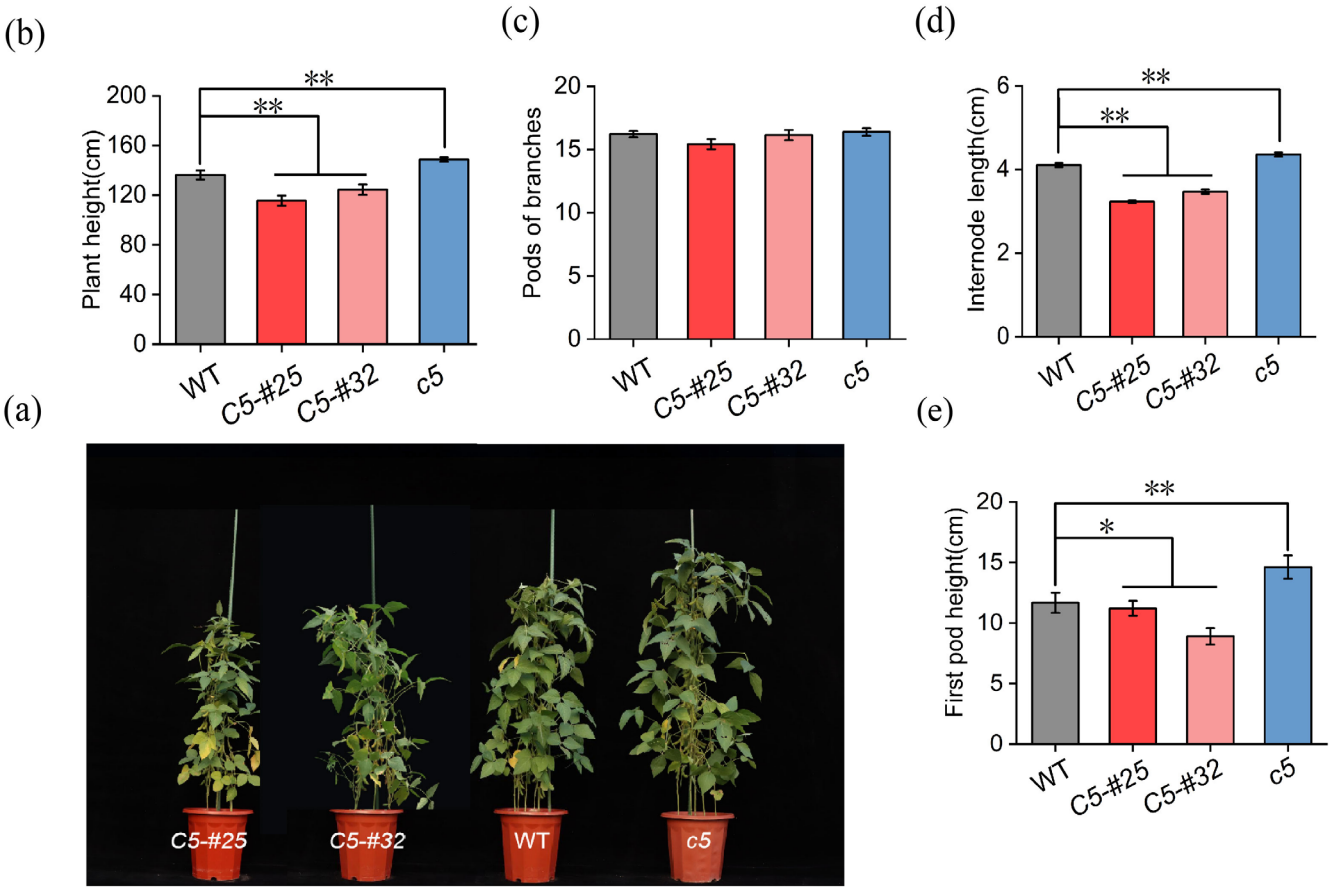
Effects of *GmNMHC5* on plant height‐related traits. (a) Phenotypes of *GmNMHC5* transgenic and WT soybean plants. (b) Plant height; (c) Number of main stem nodes; (d) Internode length; (e) First pod height. Samples: *Gmnmhc5* (*c5*), OE*GMNMHC5*‐#25 (*C5‐#25*), and OE*GMNMHC5*‐#32 (*C5‐#32*). *p < 0.05; **p < 0.01.

Phenotypic analysis revealed significant architectural alterations among the different lines. *Gmnmhc5* plants exhibited a 9% increase in plant height compared to the WT, while *GmNMHC5* overexpression lines OE*GmNMHC5*‐#25 and OE*GmNMHC5*‐#32 showed 15% and 9% reductions, respectively (Figure 2a,b). These height variations were primarily attributed to changes in internode length and lower first pod height, rather than modifications in main stem node number, which remained statistically invariant across genotypes (*p* > 0.05) (Figure 2c–e). Specifically, *Gmnmhc5* plants displayed 6% longer internodes and 25% elevated first pod height relative to the WT. Conversely, OE*GmNMHC5*‐#25 and OE*GmNMHC5*‐#32 exhibited 21% and 16% reductions in internode length, accompanied by 4% and 24% decreases in first pod height, respectively.

### 3.4 Effects of *GmNMHC5* on Soybean Seed Morphology

**FIGURE 3:**
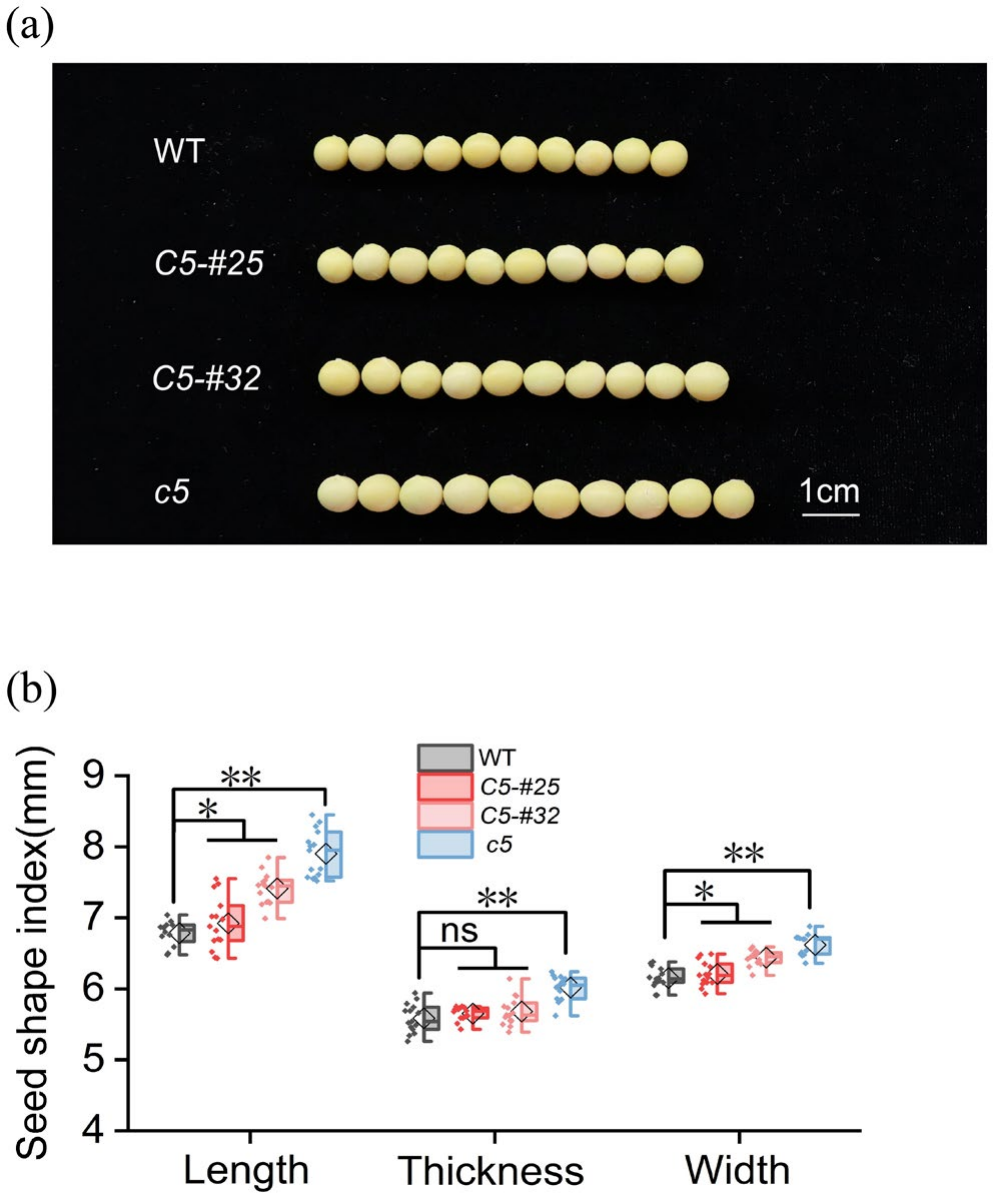
Effects of *GmNMHC5* on soybean seed size. (a) Soybean seed morphology diagram. (b) Seed length, width, and thickness. ns, not significant. Samples: *Gmnmhc5* (*c5*), OE*GMNMHC5*‐#25 (*C5‐#25*), and OE*GMNMHC5*‐#32 (*C5‐#32*). *p < 0.05; **p < 0.01.

To investigate the effect of *GmNMHC5* on seed morphology, we measured the length, width, and thickness of mature seeds from each line (Figure 3a,b). Compared to the WT, *Gmnmhc5* seeds exhibited significant increases in length (17%), width (8%), and thickness (8%). In contrast, OE*GmNMHC5*‐#25 showed no significant variations in seed dimensions (*p* > 0.05), while OE*GmNMHC5*‐#32 displayed moderate increases in length (9%) and width (5%) without significant changes in thickness.

### 3.5 Effects of *GmNMHC5* on Photosynthetic Characteristics in Soybean

**FIGURE 4:**
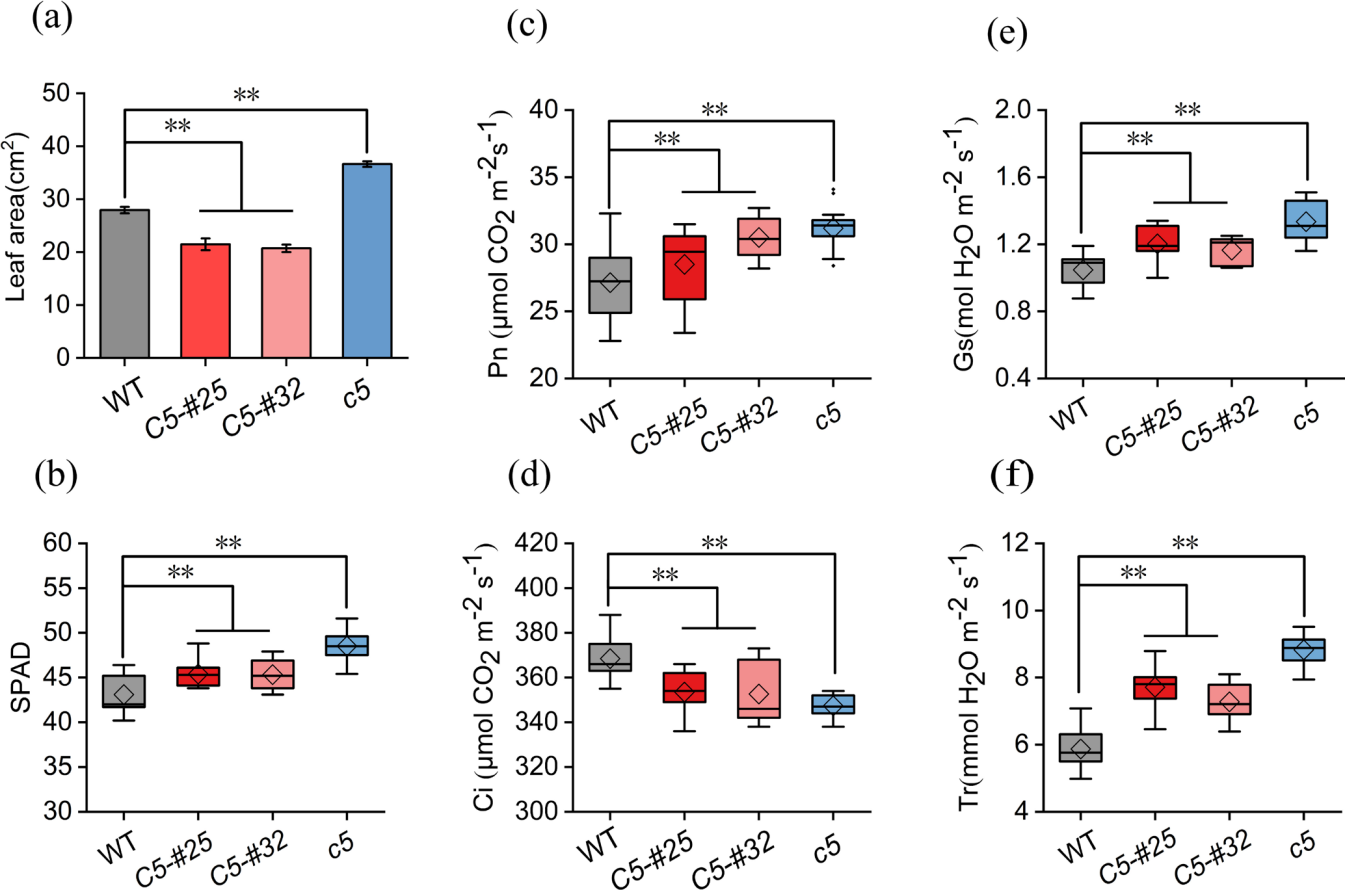
Effects of *GmNMHC5* on photosynthetic performance and SPAD value of leaves. (a) Leaf area. (b) SPAD. (c) Net photosynthetic rate. (d) Intercellular CO_2_ concentration (Ci). (e) Stomatal conductance (Gs). (f) Transpiration rate (Tr). Samples: *Gmnmhc5* (*c5*), OE*GMNMHC5*‐#25 (*C5‐#25*), and OE*GMNMHC5*‐#32 (*C5‐#32*). **p < 0.01.

Significant alterations in leaf physiological parameters were observed across the lines. *Gmnmhc5* plants exhibited a 31% increase in leaf area relative to WT, whereas *GmNMHC5* overexpression lines OE*GmNMHC5*‐#25 and OE*GmNMHC5*‐#32 showed 23% and 26% reductions, respectively (Figure 4a). Chlorophyll content, as indicated by SPAD values, increased by 13% in *Gmnmhc5* and 5% in overexpression lines compared to the WT (Figure 4b). Photosynthetic performance analysis revealed enhanced gas exchange parameters in *Gmnmhc5*, including 15% higher Pn (Figure 4c), 50% elevated Tr (Figure 4f) and 45% increased Gs (Figure 4e), accompanied by a 6% reduction in Ci (Figure 4d). Similarly, both overexpression lines demonstrated significant improvements in Pn, Tr, and Gs relative to the WT, with corresponding decreases in Ci, suggesting improved photosynthetic efficiency and stomatal regulation across transgenic genotypes.

### 3.6 Effects of *GmNMHC5* on Aboveground Dry Matter Weight and Nitrogen Content in Soybean

**FIGURE 5:**
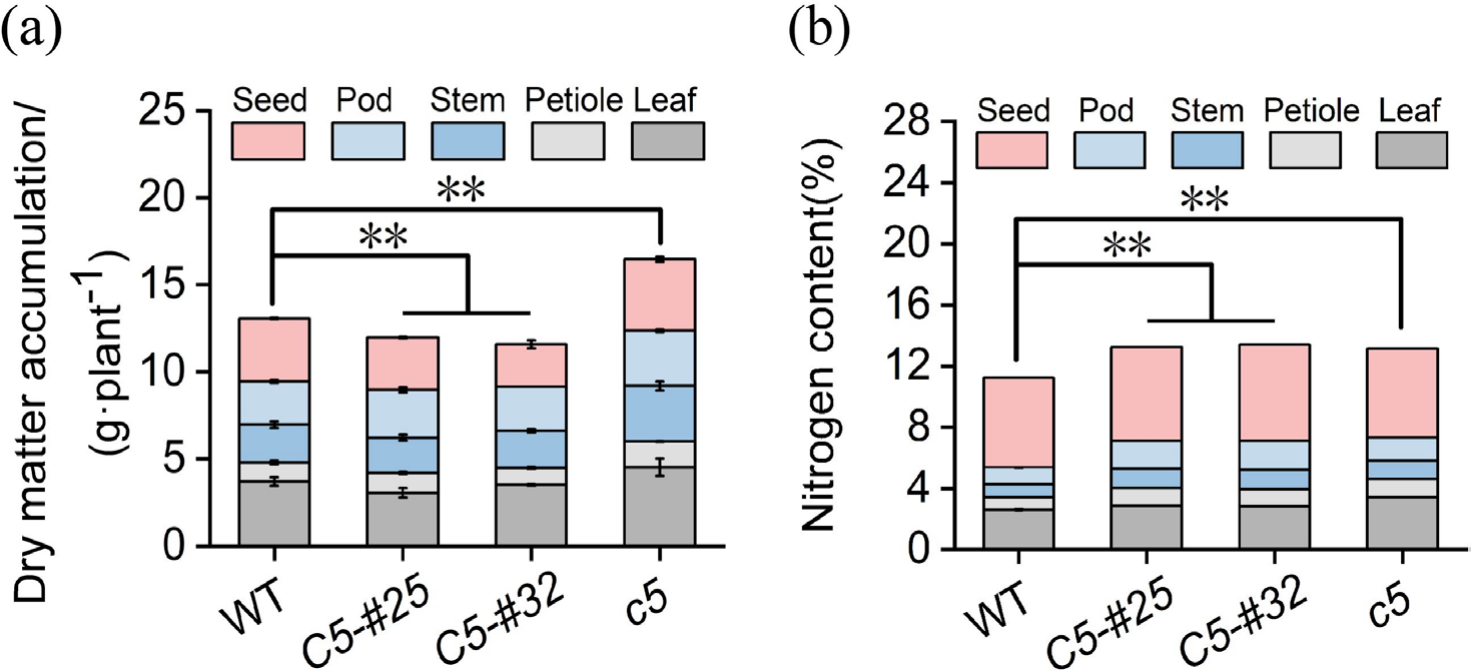
Effects of *GmNMHC5* on dry matter and nitrogen content in aboveground tissues. (a) Above‐ ground dry matter accumulation. (b) Above‐ground nitrogen content accumulation. Samples: *Gmnmhc5* (*c5*), OE*GMNMHC5*‐#25 (*C5‐#25*), and OE*GMNMHC5*‐#32 (*C5‐#32*). **p < 0.01.

*Gmnmhc5* plants exhibited 17% higher total aboveground biomass relative to the WT, driven by elevated dry weights in leaves (22%), petioles (35%), stems (46%), pods (28%), and seeds (15%). Conversely, *GmNMHC5* overexpression lines OE*GmNMHC5*‐#25 and OE*GmNMHC5*‐#32 showed 12% reductions in total biomass, with decreases in leaf (18% and 5%), stem (7% and 2%), and seed (17% and 33%) weights, respectively (Figure 5a). *Gmnmhc5* displayed 30%–53% increases in nitrogen content across vegetative (leaves, petioles, stems) and reproductive (pods) tissues, resulting in a 19% enhancement in whole‐plant nitrogen content (Figure 5b). Overexpression lines exhibited tissue‐specific nitrogen accumulation: OE*GmNMHC5*‐#25 showed 10%–67% increases in leaves, petioles, stems, pods, and seeds, while OE*GmNMHC5*‐#32 demonstrated 8%–73% elevations in corresponding tissues. These findings suggest that *GmNMHC5* modulates both carbon assimilation and nitrogen allocation during vegetative and reproductive development.

### 3.7 Effects of *GmNMHC5* on the Carbon‐to‐Nitrogen Ratio in Leaves and Seeds of Soybean

**FIGURE 6:**
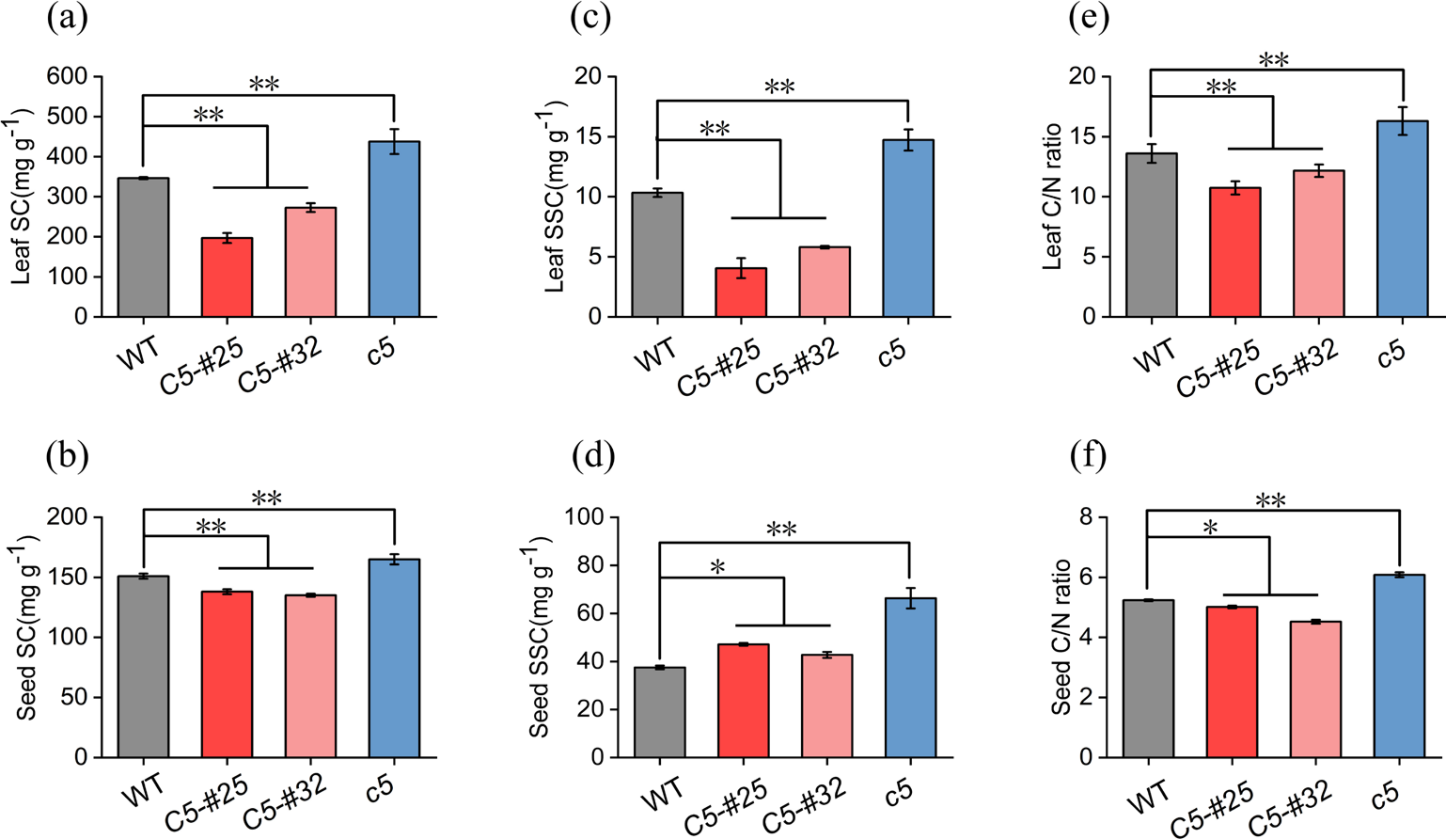
Effects of *GmNMHC5* on soluble starch content (SC), sugar content (SSC), and C/N ratio in leaves and seeds of soybean at the pod‐filling stage. (a) Starch content in leaves. (b) Starch content in seeds. (c) Soluble sugar content in leaves. (d) Soluble sugar content in seeds. (e) C/N ratio in leaves. (f) C/N ratio in seeds. Samples: *Gmnmhc5* (*c5*), OE*GMNMHC5*‐#25 (*C5‐#25*), and OE*GMNMHC5*‐#32 (*C5‐#32*). *p < 0.05; **p < 0.01.

*Gmnmhc5* plants exhibited 26% and 9% increases in leaf and seed starch content, respectively, accompanied by 42% and 77% elevations in soluble sugar levels relative to the WT (Figure 6a–d). Conversely, *GmNMHC5* overexpression lines OE*GmNMHC5*‐#25 and OE*GmNMHC5*‐#32 showed 43% and 21% reductions in leaf starch, 9% and 11% decreases in seed starch, and 61% and 44% declines in leaf soluble sugars, respectively, though seed soluble sugars increased by 26% and 14%. Compared to the WT, *Gmnmhc5* displayed 20% and 16% increases in leaf and seed C/N ratios, respectively, indicating enhanced carbon assimilation efficiency (Figure 6e,f). In contrast, overexpression lines exhibited reduced C/N ratios: OEG*mNMHC5*‐#25 showed 21% and 2% decreases in leaves and seeds, while OE*GmNMHC5*‐#32 demonstrated 11% and 14% reductions, respectively.

### 3.8 Effects of the *GmNMHC5* on Soybean Quality

Across three consecutive production seasons, the impact of *GmNMHC5* on seed quality parameters was systematically evaluated (Table 2). The overexpression lines OE*GmNMHC5*‐#25 and OE*GmNMHC5*‐#32 showed modest but consistent alterations in seed composition compared to WT averages, with oil content decreasing by 1% and 5% and protein content increasing by 1% and 3%, respectively. Despite no significant change in total protein plus oil content compared to WT, these overexpression lines had markedly lower production efficiency, exhibiting 40% and 20% decreases in oil yield, 39% and 14% reductions in protein yield, and 39% and 16% declines in total protein plus oil yield. In contrast, *Gmnmhc5* knockout plants displayed a more pronounced 6% reduction in oil content accompanied by a 6% increase in protein content, leading to a 2% improvement in total protein plus oil content. Most notably, the *Gmnmhc5* mutant maintained near‐WT oil yield levels (1% reduction) while achieving a 12% increase in protein yield, resulting in an 8% overall enhancement in total protein plus oil yield compared to the WT.

**TABLE 2:** Effects of the *GmNMHC5* on soybean quality in three growing seasons (2022–2024).

	Cultivar	Oil content(%)	Protein content(%)	Protein plus oil content (%)	Protein plus oil (g plant^−1^)
2022	WT	22.25 ± 0.12 a	38.97 ± 0.33 b	61.22 ± 0.32 ab	4.84 ± 0.03 b
	OE*GmNMHC5*‐#25	22.22 ± 0.11 a	39.09 ± 0.29 b	61.31 ± 0.30 ab	2.59 ± 0.01 c
	OE*GmNMHC5*‐#32	20.32 ± 0.18 c	40.56 ± 0.08 a	60.89 ± 0.22 b	3.48 ± 0.01 b
	*Gmnmhc5*	21.15 ± 0.17 b	40.96 ± 0.21 a	62.11 ± 0.14 a	5.25 ± 0.01 a
2023	WT	21.97 ± 0.32 a	37.92 ± 0.47 c	59.89 ± 0.37 b	4.93 ± 0.02 b
	OE*GmNMHC5*‐#25	21.54 ± 0.03 ab	38.24 ± 0.15 bc	59.78 ± 0.18 b	4.42 ± 0.01 d
	OE*GmNMHC5*‐#32	21.15 ± 0.22 b	39.14 ± 0.38 b	60.29 ± 0.47 b	4.80 ± 0.04 c
	*Gmnmhc5*	21.19 ± 0.20 b	40.75 ± 0.17 a	61.93 ± 0.26 a	5.03 ± 0.02 a
2024	WT	21.85 ± 0.15 a	38.79 ± 0.30 b	60.64 ± 0.20 b	16.01 ± 0.05 b
	OE*GmNMHC5*‐#25	21.88 ± 0.08 a	38.88 ± 0.17 b	60.76 ± 0.14 b	6.16 ± 0.01 d
	OE*GmNMHC5*‐#32	21.56 ± 0.20 a	38.94 ± 0.24 b	60.49 ± 0.09 b	13.46 ± 0.02 c
	*Gmnmhc5*	19.98 ± 0.23 b	41.42 ± 0.29 a	61.40 ± 0.29 a	17.92 ± 0.09 a

*Note:* Values with the same letter are not different at *p* < 0.05 within the same parameter. Significance analysis of yield components was performed among WT and *GmNMHC5* mutant lines within the same year.

### 3.9 Correlation Analysis

**FIGURE 7:**
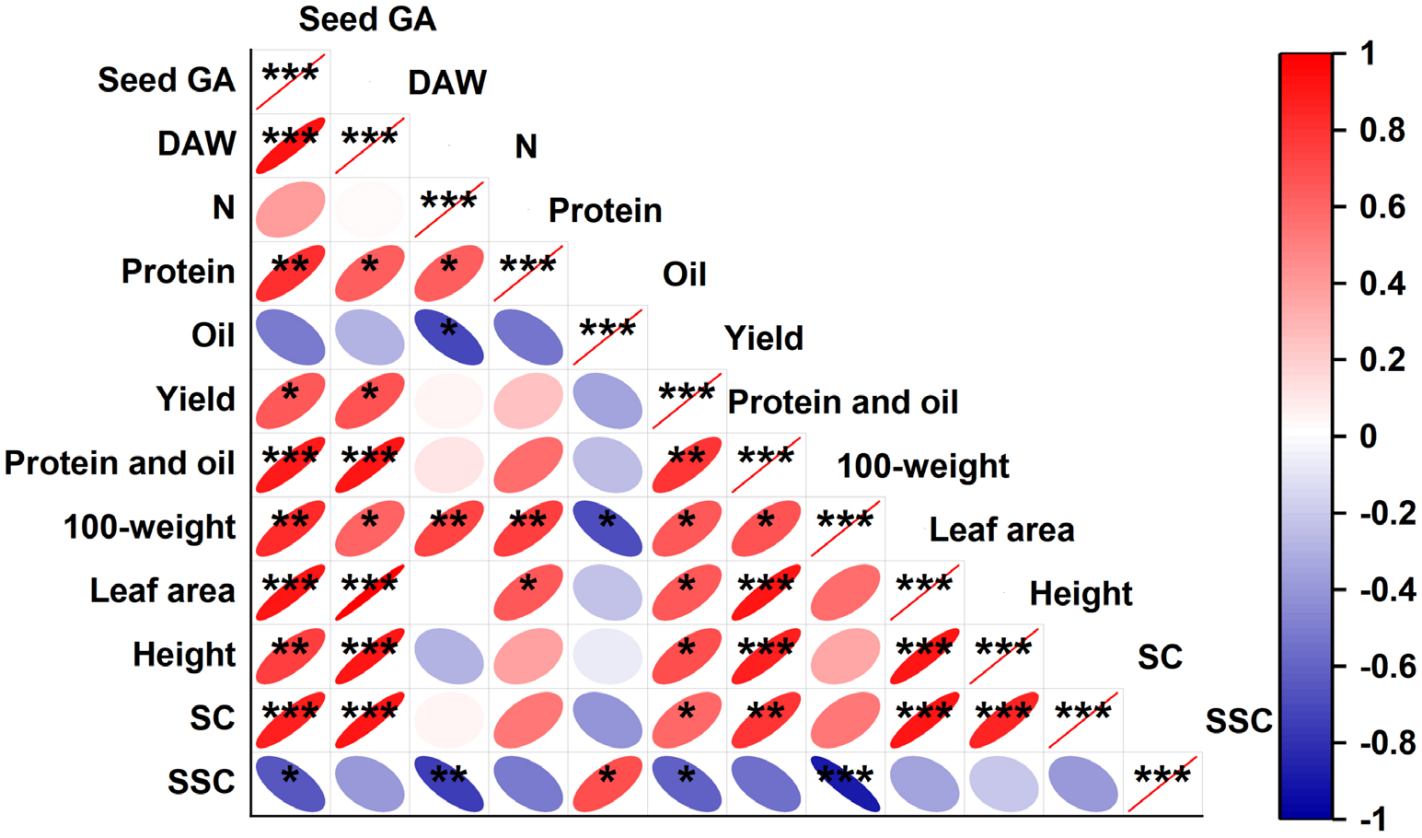
Pearson correlation analysis of the relationships among seed GA content, dry matter weight (DAW), nitrogen content (N), protein, oil, yield, protein and oil content, 100‐seed weight, leaf area, height, starch content (SC), sugar content (SSC). The roundness of the ellipses represents the significance level, with fewer circular shapes indicating higher significance. The color indicates positive or negative correlations. *p < 0.05; **p < 0.01; ***p < 0.001.

Correlation analysis revealed that seed GA content showed strong positive associations with key agronomic traits (Figure 7), including aboveground biomass (*r* = 0.93), protein content (*r* = 0.81), total protein and oil yield per plant (*r* = 0.89), 100‐seed weight (*r* = 0.82), leaf area (*r* = 0.91), plant height (*r* = 0.76), and starch content (*r* = 0.87). Furthermore, GA levels demonstrated a significant positive correlation with single‐plant yield (*r* = 0.65) but a negative correlation with soluble sugar content (*r* = −0.65) and oil content (*r* = −0.5), suggesting that GA‐mediated regulation integrated carbon partitioning and nitrogen utilization to optimize yield formation.

### 3.10 *GmNMHC5* Regulates the Expression of Genes Related to Carbon and Nitrogen Metabolism in Soybean

**FIGURE 8:**
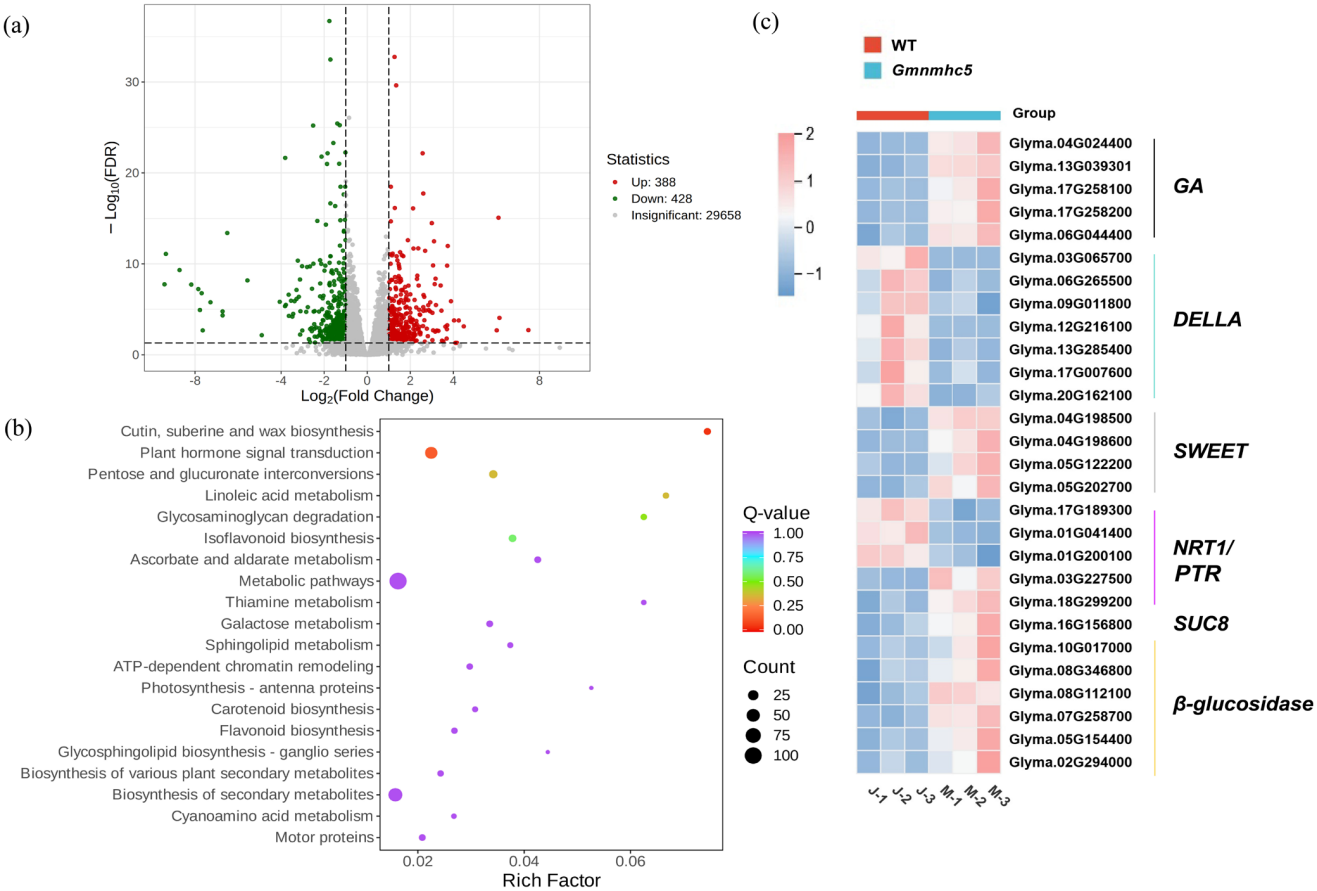
Transcriptomic analysis of carbon and nitrogen metabolism‐related genes regulated by *GmNMHC5*. (a) Volcano plot of gene expression in *Gmnmhc5* versus WT seeds at the seed‐filling stage, with red and green dots indicating upregulated and downregulated genes, respectively. (b) Kyoto Encyclopedia of Genes and Genomes (KEGG) enrichment analysis of DEGs for *Gmnmhc5* versus the WT. (c) Heatmap of carbon and nitrogen metabolism‐related DEGs in *Gmnmhc5* versus WT seeds at the filling stage, with red and blue indicating upregulated and downregulated genes, respectively. WT (Jack, J), *Gmnmhc5* (Mutant, M). DELLA, Negative regulatory proteins of gibberellin signaling; GA, Precursor protein‐coding genes for gibberellin biosynthesis; NRT1/PTR, Nitrate transporter proteins; SUC8, Sucrose transporter protein; SWEET, Sugars will eventually be exported transporter.

To elucidate the molecular mechanisms underlying *GmNMHC5*‐mediated regulation of seed development through carbon–nitrogen metabolic networks, we conducted RNA sequencing and comparative transcriptomic analysis of developing seeds from WT and *Gmnmhc5* plants (NCBI, PRJNA1244565). Differential expression analysis identified 816 differentially expressed genes (DEGs), with 428 downregulated and 388 upregulated in *Gmnmhc5* relative to WT (Figure 8a). Functional annotation revealed predominant enrichment in carbohydrate metabolism, linoleic acid metabolism, and isoflavonoid biosynthesis pathways (Figure 8b). Notably, 5 GA biosynthetic genes were upregulated in *Gmnmhc5*, while two DELLA repressors (*GAI2* and *DWARF8*) showed significant downregulation (Figure 8c). To clarify the relationship between *GmNMHC5* and carbon–nitrogen metabolism, the expression profiles of sugar transporter genes were analyzed. In *Gmnmhc5* seeds, significant upregulation was observed for sucrose transporters (*SUC8*) and SWEET family members (*SWEET5*, *SWEET10*, *SWEET11*, *SWEET15*), accompanied by increased expression of five β‐glucosidase genes involved in starch catabolism, indicating enhanced carbohydrate mobilization capacity. Nitrogen transporter analysis revealed selective regulation within the NRT1/PTR family: *PTR6*, *PTR14*, and *PTR21* were downregulated, while *PTR19* and *PTR49* showed increased expression, suggesting *GmNMHC5*‐mediated modulation of nitrate transport. Other nitrogen‐related transporters (ammonium transporters, amino acid transporters) remained largely unchanged, highlighting the specificity of *GmNMHC5*'s regulatory effects on nitrate assimilation pathways. These molecular signatures corroborated the role of *GmNMHC5* as a negative regulator of GA signaling and highlighted its integrative function in coordinating carbon–nitrogen metabolic through differential regulation of sugar and nitrate transporters.

## 4 Discussion

### 4.1 *GmNMHC5* Negatively Regulated GA Pathway in Soybeans

This study systematically elucidated the spatiotemporal regulatory mechanisms of *GmNMHC5* on GA metabolic networks at the organ‐specific level. Precise quantification of GA across organs revealed that *GmNMHC5* knockout induced gradient accumulation patterns of bioactive GAs (GA_1_ and GA_3_) in leaves, pod walls, and seeds, while specific enrichment of GA precursors GA_53_ in seeds, suggesting organ‐specific metabolic channeling functions. Transcriptomic profiling further uncovered a dual regulatory mechanism: derepression of GA biosynthetic genes to enhance GA production and downregulation of DELLA repressors such as *GAI* and *DWARF8* to amplify GA signaling responsiveness (Wang, Wang, et al. 2022; Cassani et al. 2009; Boccaccini et al. 2014). These findings agreed with the previously identified GmNMHC5‐GmGAI protein interaction (Boccaccini et al. 2014), collectively explaining systemic GA signal enhancement in mutants. Phenotypically, elevated GA levels optimized plant architecture through increased internode elongation, plant height, and leaf expansion, thereby improving light interception efficiency and photosynthetic carbon assimilation. This regulatory pattern verified the GA‐mediated plant height control mechanism observed in rice, maize, and wheat (Li et al. 2019; Hu et al. 2017; Pandey et al. 2015). Conversely, extreme GA suppression in overexpression lines triggered carbon metabolic dysregulation, evidenced by reduced soluble sugars and starch, confirming stringent GA dose‐dependent effects consistent with previous studies (Rebetzke et al. 2012; Tang et al. 2021).

### 4.2 *GmNMHC5* Improved Soybean Seed Quality Through the GA Pathway

The most pronounced effect of *GmNMHC5* was observed in soybean seed development, where knockout of this gene significantly increased seed length, width, and thickness, resulting in visibly enlarged seeds (Figure 2). This phenotypic alteration could be closely associated with elevated soluble sugar levels in both leaves and seeds of *Gmnmhc5* mutants (Figure 6c,d), whereas overexpression lines exhibited reduced soluble sugars, suggesting a critical role of sugar transport during seed filling. In rice, *OsSWEET11* and *OsSWEET15* have been identified as key regulators of seed filling (Yang et al. 2018), while in soybeans, upregulation of *GmSWEET10a* increased seed size and oil content but reduced protein accumulation (Wang, Wang, et al. 2022). Notably, GA‐mediated regulation of sugar transporters (*ATSWEET13*, *ATSWEET14*) has been shown to influence seed development, with double mutants exhibiting reduced GA levels and altered seed morphology (Kanno et al. 2016). Similarly, in wheat, *TaGW2‐6A* negatively regulates GA biosynthesis through GA_3_‐oxidase, precisely controlling endosperm cell elongation and division during grain filling, ultimately increasing seed size (Li et al. 2017). These findings collectively demonstrate that GA influences seed development by modulating sugar transport and allocation.

Beyond seed size, GA significantly influences seed quality. *Gmnmhc5* plant exhibited a 7% increase in protein content, leading to a 2% rise in total protein and oil yield per plant (Table 2). This highlighted the potential of *GmNMHC5* manipulation for improving seed nutritional quality. Agronomic applications of GA, such as exogenous GA_3_ application during vegetative growth, have been shown to enhance stem elongation, leaf expansion, and root development in soybeans while increasing leaf area, biomass, and yield (Shan et al. 2021). Exogenous GA_3_ disrupted endogenous GA homeostasis, rapidly altering plant growth patterns (Zhao et al. 2025; Fang et al. 2021). Our study further revealed that *GmNMHC5* primarily regulated GA concentration by modulating the biosynthesis of bioactive GA_1_ and GA_3_ (Figure 1), with GA_3_ playing a decisive role in leaf, pod wall, and seed development during the R5.5 stage.

### 4.3 *GmNMHC5* Regulated the C–N Metabolism in Soybeans

The pivotal role of *GmNMHC5* in regulating soybean C–N metabolism through the GA signaling pathway was indicated in the study. In carbon metabolism, loss of *GmNMHC5* function disrupted the negative feedback regulation of GA biosynthesis, leading to elevated GA levels that promoted vegetative organ growth. This resulted in an increase in leaf area, enhancement of chlorophyll content, and improvement of photosynthetic carbon assimilation efficiency, driving the expansion of sucrose and non‐structural carbohydrate (NSC) pools (Li et al. 2019; Wang, Wang, et al. 2022; Stavang et al. 2006). Additionally, *GmNMHC5* mutation enhanced the expression of sugar transporters (SWEETs), sucrose synthases (SUS), and β‐glucosidases in leaves, optimizing carbon source synthesis and flux partitioning. For nitrogen metabolism, *GmNMHC5* knockout plants exhibit significantly improved nitrogen assimilation capacity, with elevated nitrogen accumulation across all organs. Transcriptomic data further demonstrated that *GmNMHC5* deletion promoted the transcription of nitrogen transporters (NRT1/PTR family), consistent with previous findings in wheat (Wang, Yao, et al. 2020; Sun et al. 2023). These results establish *GmNMHC5* as a key coordinator of C–N metabolic integration through GA signaling, providing a novel molecular target for simultaneous improvement of soybean yield and quality.

### 4.4 *GmNMHC5* Influenced the Yield and Quality by Regulating the Balance of C–N Allocation in Soybeans

**FIGURE 9:**
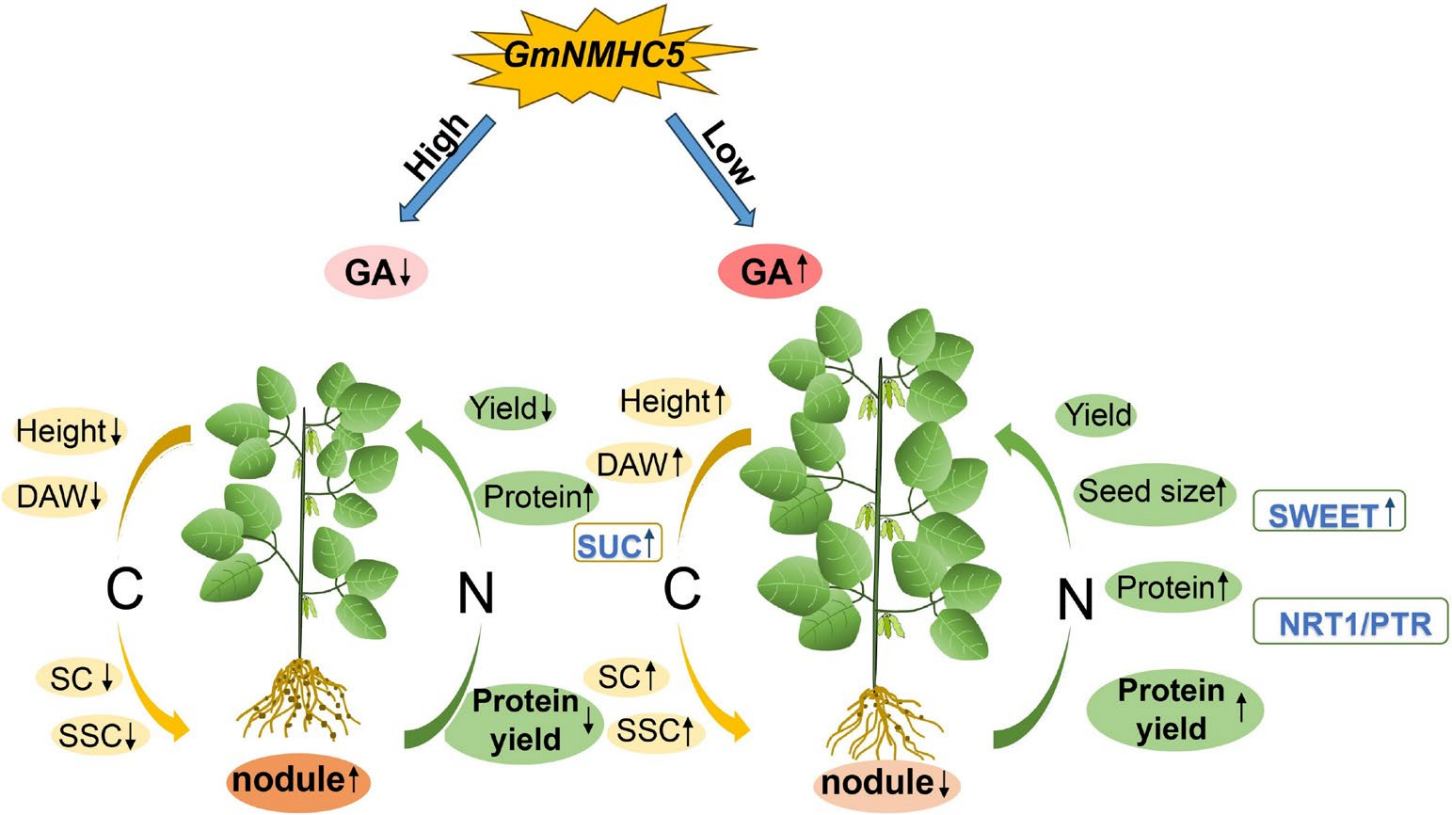
The impact of *GmNMHC5* on GA content and C–N metabolism patterns in soybean plants.

The knockout of *GmNMHC5* significantly altered soybean growth dynamics, revealing its critical role in balancing C–N allocation to optimize yield and quality. The underlying mechanisms were schematically illustrated in Figure 9. *Gmnmhc5* mutant displayed enhanced vegetative growth, including increased plant height, leaf area, and biomass. However, this improvement was accompanied by reduced reproductive efficiency, as evidenced by significant decreases in pod number and seed number per plant. The reduction in seed number was partially attributed to diminished root nodulation, as *GmNMHC5* knockout reduced nodule number and nitrogen fixation capacity through its interaction with the DELLA protein GmGAI (Wang, Wang, et al. 2022). Given the strong correlation between nodule number, nitrogenase activity, and total nitrogen content (Chu et al. 2022), the compromised nitrogen acquisition in *Gmnmhc5* likely limited the seed set, despite increased carbon assimilation.

Elevated GA levels in the *Gmnmhc5* mutant drove carbon flux redirection, with soluble sugars and starch accumulating in leaves. This enhanced carbon assimilation supported compensatory seed enlargement, offsetting the reduced seed number. Such resource reallocation highlighted *GmNMHC5*'s role as a metabolic integrator (Liu et al. 2015), fine‐tuning C–N partitioning through GA signaling to balance vegetative and reproductive growth.

Conversely, *GmNMHC5* overexpression severely restricted both vegetative and reproductive development. Reduced GA levels led to dwarfism, decreased biomass, and impaired photosynthetic product allocation, despite increased nitrogen content. While enhanced nodulation in overexpression lines potentially improved nitrogen fixation, the energy cost of nodule formation likely diverted resources from leaf expansion and photosynthetic capacity (Liu et al. 2015; Wang, Wang, et al. 2022). This imbalance resulted in a significant yield penalty, with pod number and seed number drastically reduced in overexpression lines.

To maximize soybean productivity, optimizing *GmNMHC5* expression to achieve balanced source‐sink relationships was crucial. Future research should focus on fine‐tuning *GmNMHC5* expression levels to synchronize C–N assimilation with reproductive development, engineering nodulation efficiency to minimize energy trade‐offs, and investigating *GmNMHC5*'s role in flowering regulation to address the observed reductions in pod and seed number (Wang, Wang, et al. 2020). These strategies would enable the development of soybean varieties with optimized C–N allocation, enhancing both yield and nutritional quality.

## 5 Conclusions

This study systematically elucidated GA‐mediated control of soybean quality and yield through quantification of GA derivatives (GA_1_, GA_3_, GA_4_) in source (leaves) versus sink (pod walls, seeds) tissues during the critical seed‐filling stage (R5.5 stage). Comprehensive phenotyping revealed that *GmNMHC5* knockout lines exhibited negative regulation of GA biosynthesis, leading to changes in endogenous GA levels, which in turn influenced plant height and dry matter accumulation. These alterations in GA signaling further modulated C–N metabolism, ultimately enhancing the protein yield in soybean seeds. Our findings established *GmNMHC5* as a central regulator linking phytohormone signaling with primary metabolism during seed development.

## Author Contributions

**Xinlei Chen:** data curation (lead), methodology (lead), writing – original draft (lead), investigation (lead), visualization (lead). **Wenwen Song:** methodology (equal), formal analysis (lead), writing – review and editing (lead). **Zhongfa Zhang:** investigation (supporting), visualization (supporting). **Chenchen Zhou:** visualization (supporting). **Peihang Wu:** investigation (supporting). **Shujun Wang:** formal analysis (equal), data curation (supporting). **Shi Sun:** resources (supporting). **Yupeng Zhu:** conceptualization (supporting), writing – review and editing (equal), project administration (supporting). **Cailong Xu:** conceptualization (equal), validation (lead), writing – review and editing (supporting), project administration (supporting), funding acquisition (equal). **Cunxiang Wu:** conceptualization (lead), validation (supporting), resources (lead), supervision (lead), project administration (lead), funding acquisition (lead).

## Conflicts of Interest

The authors declare no conflicts of interest.

## Supporting information


Data S1.

## Data Availability

The data presented in this study are available on request from the corresponding authors.
