# Supplementary material for: GmNMHC5 Modulates Gibberellin Homeostasis to Balance Carbon–Nitrogen Metabolism and Enhance Protein Yield in Soybean
Source: Food Sci Nutr. 2025 Aug 7;13(8):e70659. doi: 10.1002/fsn3.70659 (PMC12329567; doi:10.1002/fsn3.70659)
Supplement: Supplementary file 1 — Data S1. [file FSN3-13-e70659-s001.docx]

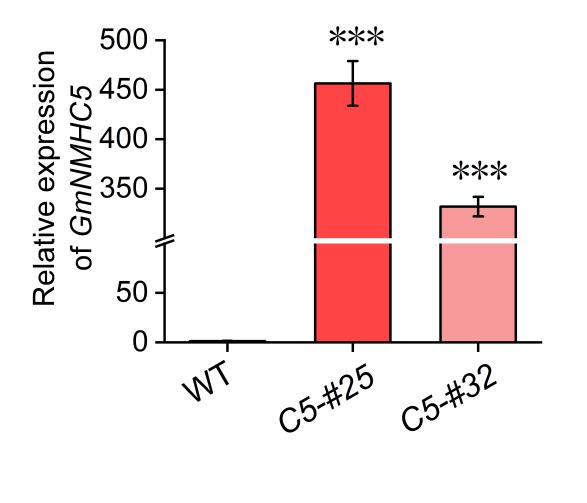


**FIGURE S1.** The relative expression of *GmNMHC5* in WT, OEGm*NMHC5*-#25 (*C5-#25*) and OE*GmNMHC5*-#32 *(C5-#32*).


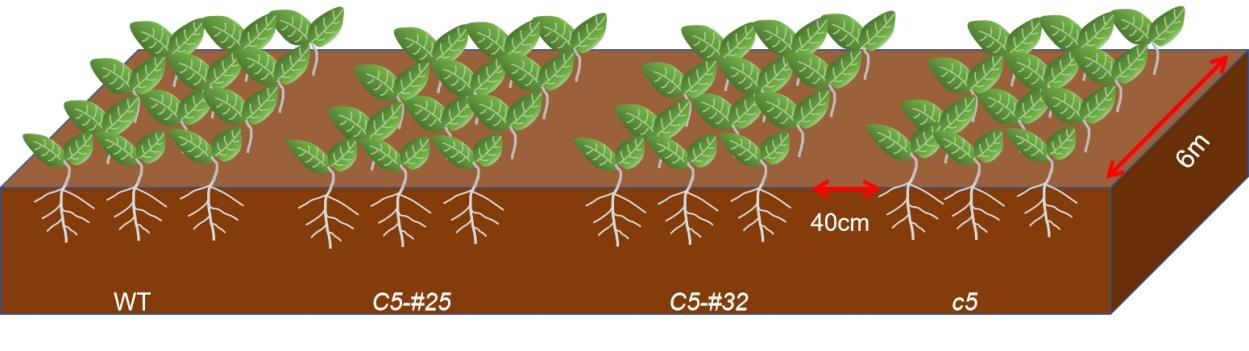


**FIGURE S2.** Field experiment design diagram. Samples: *Gmnmhc5* (*c5*), OE*GMNMHC5*-#25 (*C5-#25*), and OE*GMNMHC5*-#32 (*C5-#32*).

**TABLE S1.** The nucleotide sequences of the primers used in this study.

| **Gene** | **Gene ID** | **Primer name** | **Primer sequence (5’ to 3’)** |
| --- | --- | --- | --- |
| *GmNMHC5* | *Glyma.13G255200* | F-qG5-F | GGGTGTCCGCATGAAAAAGG |
|  |  | F-qG5-R | GGATTGGATGCCGCATTTTCT |
| *GmActin* | *Glyma.18G290800* | qGmActin-F | CGGTGGTTCTATCTTGGCATC |
|  |  | qGmActin-R | GTCTTTCGCTTCAATAACCCTA |

**TABLE S2.** GA contents (ng g^-1^) in leaves, pod walls, and seeds of WT, OE*GmNMHC5*-#25 (*C5-#25*) and OE*GmNMHC5*-#32 *(C5-#32*), *Gmnmhc5* (*c5*).

| **GA** | **Leaf** | | | | **Seed** | | | | **Pod wall** | | | |
| --- | --- | --- | --- | --- | --- | --- | --- | --- | --- | --- | --- | --- |
|  | **WT** | ***C5-#25*** | ***C5-#32*** | ***c5*** | **WT** | ***C5-#25*** | ***C5-#32*** | ***c5*** | **WT** | ***C5-#25*** | ***C5-#32*** | ***c5*** |
| **GA15** | 0.0878 | 0.0176 | 0.0171 | 0.0339 | 1.937 | 1.6123 | 1.6788 | 1.465 | 0.004 | 0.0081 | 0.0054 | 0.0414 |
| **GA34** |  |  |  |  |  |  |  |  |  |  |  |  |
| **GA29** |  |  |  |  |  |  |  |  |  | 0.5087 | 0.6142 |  |
| **GA6** |  |  |  |  | 0.1191 | 0.1659 | 0.3728 |  |  |  |  |  |
| **GA8** |  |  |  |  |  | 1.0062 | 0.9563 | 1.1705 |  |  |  |  |
| **GA7** |  |  |  |  | 0.2452 | 0.2354 | 0.2233 | 0.3199 |  |  |  |  |
| **GA24** |  |  |  |  | 0.17 | 0.2239 | 0.2256 | 0.1618 |  |  |  |  |
| **GA9** |  |  |  |  | 0.7912 | 0.7752 | 1.4121 | 1.0512 |  |  |  |  |
| **GA44** |  |  |  |  | 0.6739 | 1.461 | 1.7256 | 1.0843 |  |  |  |  |
| **GA4** |  |  |  | 0.1398 | 4.6317 | 6.1367 | 7.7359 | 5.3188 |  |  |  | 0.2809 |
| **GA53** |  |  |  |  | 34.8745 | 31.4582 | 36.8254 | 50.7398 | 5.7086 | 0.1863 |  | 0.8156 |
| **GA20** | 0.4873 | 0.5431 | 0.5841 | 0.5978 | 0.1938 | 0.2605 | 0.2618 | 0.2724 |  |  |  | 0.1734 |
| **GA12** | 0.0044 |  | 0.0212 |  | 0.0763 | 0.0506 | 0.0374 | 0.1337 | 0.039 | 0.0389 | 0.0414 | 0.1002 |
| **GA1** | 1.9135 | 0.5298 | 0.5345 | 6.7001 | 2.0978 | 3.0614 | 2.8376 | 9.8683 | 1.3141 |  |  | 20.8824 |
| **GA19** | 0.6805 |  | 1.0448 | 0.7132 | 16.8438 | 6.9587 | 6.0959 | 20.0382 | 10.8952 | 2.3922 | 3.6803 | 3.4383 |
| **GA3** | 12.7616 | 1.5794 | 1.3248 | 69.1274 | 6.691 | 2.8529 | 2.7001 | 96.6084 | 10.0792 | 9.0418 | 6.5565 | 202.9945 |
| **Total** | 15.9351 | 2.6699 | 3.5265 | 77.3122 | 69.3453 | 56.2589 | 63.0886 | 188.2323 | 28.0401 | 12.176 | 10.8978 | 228.7267 |
